# Voxel-based Histographic Analysis of the Basilar Artery in Patients with Isolated Pontine Infarction

**DOI:** 10.2463/mrms.mp.2015-0103

**Published:** 2016-02-20

**Authors:** Han Won JANG, Hui Joong LEE, Jongmin LEE, Chulho WON, Sung Won YOUN, Mun HAN, Yongmin CHANG

**Affiliations:** 1 Daekyung Radiology Clinics, Kyungpook National University Hospital, Daegu, Republic of Korea, Dongduk-ro 200 Jung-gu, Daegu 700-721, Republic of Korea; 2 Department of Radiology, Kyungpook National University Hospital, Daegu, Republic of Korea, Dongduk-ro 200 Jung-gu, Daegu 700-721, Republic of Korea; 3 Department of Control Instrumentation Engineering, Kyungil University; 4 School of Electrical Engineering and Computer Sciences, Kyungpook National University; 5 Department of Radiology, Catholic University of Daegu, Medical Center; 6 Department of Medical & Biological Engineering, Kyungpook National University; 7 Department of Molecular Medicine, Kyungpook National University

**Keywords:** histogram, MRI, atherosclerosis, stroke, basilar artery

## Abstract

**Background and Purpose:**

The signal information per voxels of magnetic resonance imaging (MRI) for vessel wall could reflect the pathologic features of atherosclerotic vessels. The aim of this study is to evaluate the usefulness of magnetic resonance voxel-based histogram (VBH) of atherosclerotic basilar artery in patients with isolated pontine infarctions (PIs).

**Materials and Methods:**

Wall and lumen of basilar artery were segmented from high resolution MR of 42 patients with isolated PI and 10 normal volunteers. VBHs were obtained after normalization by dividing the intensity of segmented wall with the intensity of non-infarcted area of pons. The variables of VBH included area (A), mean signal intensity (SI), standard deviation (SD), kurtosis (K), and skewness (SK) and area stenosis [AS; A^wall^/(A^wall^ + A^lumen^)] were compared according to the MRI-modified American Heart Association (AHA) atherosclerotic plaque schema, and between the subgroups of PI (lacunar: LPI and paramedian: PPI).

**Results:**

According to the MRI-modified AHA atherosclerotic plaque schema, A^wall/T_1_^ (mean area of wall on T_1_-weighted MRI), SI^wall/T_1_^, SD^wall/T_1_^, SK^wall/T_1_^, K^wall/T_1_^, A^lumen/T_1_^, and AS^T_1_^ showed statistical differences. AHA IV–VII showed higher A^wall/T_1_^, SI^wall/T_1_^, and AS^T_1_^ than normal control. PPI showed statistical differences in A^wall/T_1_^, SI^wall/T_1_^, SK ^wall/T_1_^, and A^wall/T_2_^ than those of normal control after post hoc test, whereas LPI in A^wall/T_1_^ and A^wall/T_2_^ (*P* < 0.05, Kruskal-Wallis test, Dunnett T3 procedure).

**Conclusions:**

VBH analysis can provide the quantitative information with regard to volume as well as composition of the atherosclerotic plaque in the basilar artery. The difference in patterns of VBH might be further useful in characterizing PIs with presumably different pathogenesis.

## Introduction

An isolated pontine infarction (PI) can be subdivided into a paramedian pontine infarction (PPI) or a lacunar pontine infarction (LPI), depending upon the involvement of the ventral surface of the pons. Several autopsy and neuroimaging studies have supported this way of classification,^1–11^ and it would be of clinical significance to identify one from another since the rates of recurrence, disability, and mortality are different between PPI and LPI.^12^ It has been known that a PPI is caused by a “large vessel disease” resulting in atherosclerotic occlusion of the orifice of perforating artery.^1,2^ On the other hand, LPI or small deep PI is related to small lipohyalinosis or arteriosclerotic occlusion of a small perforating vessel itself, as a result of “small vessel disease”. According to Erro et al., patients with PPI have a higher frequency of basilar artery stenosis and they have a worse prognosis than patients with LPI.^7^

The assessment of basilar artery stenosis can be made with several vascular imaging modalities including digital subtraction angiography, computed tomography angiography (CTA), and magnetic resonance angiography (MRA).^13^ However, such imaging modalities have presented simple degree of luminal narrowing, which have drawbacks in characterizing atherosclerotic plaque. Especially during the earlier stage of atherosclerosis development, the vessel wall can remodel to expand outward, maintaining a normal arterial lumen despite plaque growth, referred as “positive remodeling.”

In this respect, high-resolution magnetic resonance (HRMR) imaging has emerged as a promising technique to evaluate atherosclerotic disease in vivo.^14–16^ HRMR is a useful tool for imaging the vessel wall and atherosclerotic plaque of intracranial and extracranial arteries even in cases without narrowing of the lumen.^14,16,17^ Klein et al. reported that HRMR detected basilar artery atherosclerosis in 42% of patients with isolated PI and normal appearing basilar artery on time-of-flight (TOF) MRA. An atherosclerotic plaque was detected on HRMR in 61.5% of patients with PPI, whereas basilar lumen narrowing was shown on TOF MRA in only 35%. These suggested that HRMR was more sensitive than angiography in detecting atherosclerosis, and PPI could be mostly caused by large artery disease.^16^ Interestingly, another recent study using HRMR had shown that LPI or a penetrating artery occlusion may also occur secondarily to basilar artery atherosclerosis,^15^ suggesting that basilar atherosclerotic plaque could be in fact much more frequent in LPI than previously thought. However, the previous studies using HRMR were performed with visual assessment until recently. When atherosclerotic change began, even the luminal size or area of vessel wall was similar to that of normal vessel; the component of vessel could be different.

We assumed that these conflicting results might be resolved by voxel-based quantitative assessment. HRMR provides excellent boundary definition, for example, in the basilar artery-CSF border and the blood-intima border, which make it possible to segment and to calculate the cross-sectional areas of basilar artery. In addition, as each voxel of HRMR of atherosclerotic vessels represents various tissue components of atherosclerotic plaque and wall according to the physical property of the corresponding tissue, voxel-based analysis could be expected to present even minute differences. Here we explore whether voxel-based histogram (VBH) analysis of basilar artery explain the cause of PI. When atherosclerotic change began, even the luminal size or area of vessel wall was similar to that of normal vessel; the component of vessel could be different. Kurtosis and skewness of VBH could reflect the change of component. The aim of this study is to evaluate the usefulness of VBH for HRMR of atherosclerotic basilar artery in patients with isolated PI.

## Materials and Methods

### Patient characteristics

Institutional review board approval was obtained, and the requirement for informed consent was waived for this retrospective study and written informed consents were obtained from normal control volunteers. Between January 2007 and December 2011, 42 consecutive patients (M:F = 25:17 age range, 45–85 years; mean age ± standard deviation, 65.1 ± 10.6 years) with an acute isolated PI were included for this study. In order to exclude artery-to-artery embolization and cardiogenic embolic infarct, the patients were excluded if they presented with extensive infarcts involving the neighboring midbrain and medulla oblongata, had previous cerebellar and supra tentorial infarcts, or also had a potential source of a cardiogenic embolism such as atrial fibrillation, valvular heart disease, or congestive heart failure. Demographic features and risk factors were recorded including hypertension (defined as receiving medication for hypertension or blood pressure >140 mmHg on systolic and >90 on diastolic pressure on repeated measurements), diabetes mellitus (defined as receiving medication for diabetes mellitus or fasting blood sugar is above 126 mg/dl or postprandial 2 hour above 200 mg/dl), and their current cigarette smoking status. To assess the clinical status, Glasgow Coma Scale (GCS), National Institutes of Health Stroke Scale (NIHSS) score was measured at the time of patients’ admission. The modified Rankin Scale and duration of admission were measured at the time of their discharge to assess the clinical outcome.

### MR protocol

A 1.5-Tesla MRI unit (Intera, Philips Medical Systems, The Netherlands) was utilized for the study. All the patients underwent magnetic resonance imaging (MRI) including diffusion-weighted imaging (DWI), apparent diffusion coefficient (ADC) measurement, HRMR, and 3D-TOF MRA within 7 days after the symptom onset. MRA using 3D-TOF technique [(repetition time (TR): 18 msec, echo time (TE): 6.9 msec, flip angle: 20°, field of view (FOV): 18 cm × 15 cm, matrix: 236 × 512, slice thickness: 1.3 mm, number of excitation (NEX): 1] was performed to evaluate the basilar arteries. The HRMR comprised T_1_-weighted images (T_1_WIs; TR: 532 msec, TE: 10 msec, FOV: 80 mm × 80 mm, thickness: 2 mm, slice gap: 0.4 mm, NEX: 14, matrix: 512 × 512, resolution: 6.4 pixels/mm, pixel size: 0.02414 mm^2^, scan time: 64 sec) and T_2_-weighted images (T_2_WIs; TR: 2500 msec, TE: 70 msec, FOV: 80 mm × 80 mm, thickness: 2 mm, NEX: 12, matrix: 512 × 512, resolution: 6.4 pixels/mm, pixel size: 0.02414 mm^2^, scan time: 256 sec). Ten volunteers (5 men, 5 women, mean age 27 years, and age range 16–35 years) also undertook HRMR in order to analyze the normal HRMR findings of basilar artery.

### Visual assessment of MRI

**Fig. 1.**
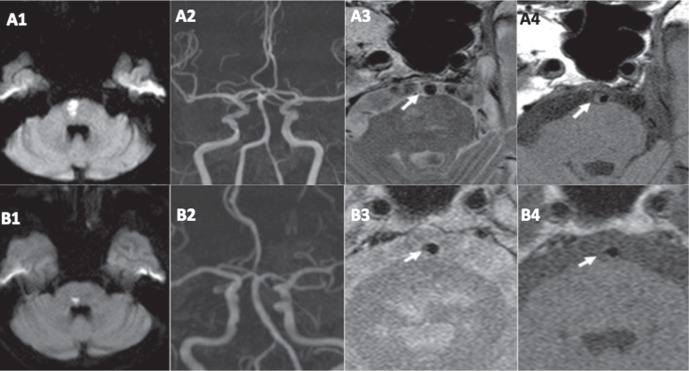
High resolution MR for patient with isolated paramedian pontine infarction (**A**: paramedian pontine infarction; **B**: lacunar pontine infarction; 1: diffusion-weighted image; 2: TOF MRA; 3: T_2_-weighted image; 4: T_1_-weighted image). TOF MRA (A2) shows slight narrowing of basilar artery in a patient with paramedian pontine infarction which is seen as atherosclerotic plaque (arrow). TOF MRA (B2) shows normal caliber of basilar artery in a patient with lacunar pontine infarction, whereas atherosclerotic plaque are seen on T_1_- (B3) and T_2_- (B4) weighted MRI (arrow). MRI, magnetic resonance imaging; MRA, magnetic resonance angiography; TOF, time-of-flight.

All MR images were interpreted on a picture archiving and communications system workstation (PiViewStar; Infiniti, Seoul, Korea) in order to classify PI by two experienced neuroradiologists (H.W.J and H.J.L with 10 and 11 years of experience in Neuroradiology, respectively) independently (Fig. 1). Isolated PIs were classified into PPI and LPI, depending on whether infarct involves the ventral surface of the pons. Two radiologists (H.W.J and H.J.L) independently reviewed MR image for the quantitative and qualitative analysis of basilar artery in order to detect the presence and degree of basilar artery stenosis. The locations of plaque were considered to correspond to the relevant PI in all the patients. The morphology of plaque on wall was classified according to the modified American Heart Association (AHA) atherosclerotic plaque schema based on MRI.^18^ AHA Type I & II were regarded as absence plaque on the basis of technical limitations of MRI resolution for basilar artery. AHA Type III was defined as a slight diffuse or eccentric thickening of the wall containing pools of extracellular lipid. AHA Type IV–VIII defined by MR may have high or iso-signal intensity on T_1_WI, and varied signal intensities on T_2_WI. AHA Type III is classified as such when the criteria for Type IV–V, VI, and VII are excluded. If there was disagreement between two observers, the radiologists reached a consensus after discussion. Shortest and longest inner diameters were measured on HRMR, as well as outer. The compromised perforating artery was defined if atheroma was located at the posterior portion of basilar artery.

### VBH

**Fig. 2.**
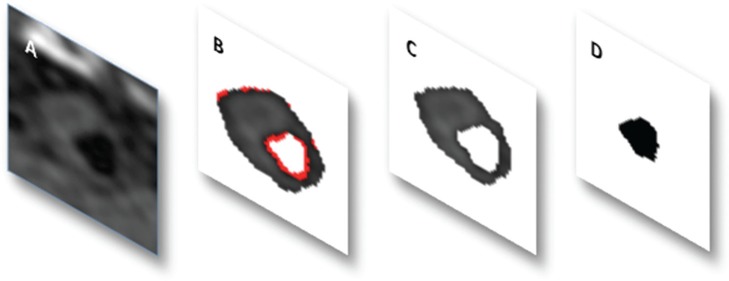
Segmentation of vessel wall and lumen, Software: ImageJ1.47q (NIH, USA); (**A**) raw data, (**B**) segmentation with ROI and threshold technique, (**C**) vessel wall, and (**D**) lumen. ROI, region of interest.

Each T_1_- and T_2_WIs were segmented into vessel wall and lumen using manual region of interest technique, followed by threshold technique to tease out the wall and lumen with Image J program to give same condition for each image and reproducible later on the same data (1.47q, National Institutes of Health, Bethesda) (Fig. 2). By multiplying the mask image (signal intensity of wall = 1, others = 0) to segmented images, we suppressed artifact such as flow-related enhancement. To normalize the MR signal intensity, the intensity of segmented wall image was divided by the intensity of non-infarcted area of pons. And then, the numbers of voxels, area, skewness, kurtosis, and normalized signal intensity of segmented vessel wall and lumens were calculated using the program. VBH for vessel and lumen were produced (Bins = 100). The pixel information, including area (A), mean (SI), and standard deviation (SD) of signal intensity, kurtosis (K), and skewness (SK), were compared, according to the MRI-modified AHA atherosclerotic plaque schema and subgroups of PI. Area stenosis [A^wall^/(A^wall^ + A^lumen^)] were calculated as to evaluate the atherosclerotic degrees of vessels.

### Statistical analysis

Statistical analysis was performed with SPSS v13.0 for Windows (SPSS; Chicago, Illinois, USA). Fishers’ exact test was applied to examine the difference in categorical variables, because the sample size was small. For the comparison of atherosclerotic parameters, statistical significance was evaluated by using the Kruskal-Wallis test. For multiple comparisons according to atherosclerosis and PI, post hoc tests (Dunnett T3 procedure) were performed. Statistical significance was set at *P* < 0.05.

## Results

There were hypertensions in 29 (69.1%), diabetes mellitus in 18 (42.9%), cigarette smoking in 18 (42.9%), and hypercholesterolemia in 10 (23.8%) patients. The initial NIHSS score ranged from 1 to 18 (mean = 4.37 ± 3.94). As depicted on DWI, 16 (38.1%) of 42 patients had LPI and 26 (61.9%) had PPI. In comparison between PPI and LPI, there was no statistical difference of demographic data except age and initial NIHSs (Table 1).

Eight patients (19.0%) without identified plaques on basilar artery were graded as AHA Type I & II. Eighteen patients (42.9%) with slight diffuse or eccentric thickening of the wall were graded AHA Type III. Sixteen patients (38.1%) were graded as AHA Type IV–VIII. The inner diameter of patients with basilar artery stenosis showed narrow lumen than that of patients without basilar artery stenosis (*P* < 0.001). On projection image of TOF MRA, one of AHA Type I & II, four of AHA Type III, and six of AHA Type IV–VIII showed atherosclerotic plaque (*P* = 0.053), (Table 2).

**Fig. 3.**
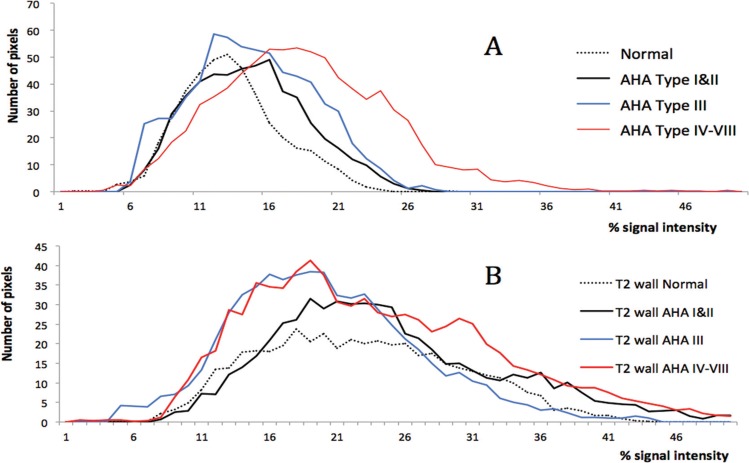
T_1_ (**A**) and T_2_ (**B**) VBHs of wall according to the MRI-modified AHA atherosclerotic plaque schema (normal: dot; AHA Type I & II solid; AHA Type III: blue; AHA Type IV–VIII; red). On T_1_ VBH for vessel wall, the mean was shifted to right side according to the MRI-modified AHA atherosclerotic plaque schema. AHA, American Heart Association; MRI, magnetic resonance imaging; VBH, voxel-based histogram.

To compare the data of VBH with normal controls, 10 volunteers undertook HRMR examinations of basilar artery using the same protocols as the patients with PI. The mean inner luminal diameter was 3.21 ± 0.31 mm, and the outer diameter was 4.62 ± 0.47 mm. On T_1_ VBH for vessel wall, the mean was shifted to right side according to the MRI-modified AHA atherosclerotic plaque schema (Fig. 3).

In comparison of the parameters of VBH according to the MRI-modified AHA atherosclerotic plaque schema, A^wall/T_1_^ (mean area of wall on T_1_WI), SI^wall/T_1_^, SD^wall/T_1_^, SK^wall/T_1_^, K^wall/T_1_^, A^lumen/T_1_^ and the AS^T_1_^ showed statistical differences (*P* < 0.05, Kruskal-Wallis test). Although, post hoc test (Dunnett T3 procedure) showed no statistical differences between normal control and AHA Type I & II, A^wall/T_1_^, SI^wall/T_1_^, SD^wall/T_1_^, SK^wall/T_1_^, K^wall/T_1_^, A^lumen/T_1_^, AS^T_1_^, and AS^T_2_^ were significantly different to differentiate AHA Type III and AHA Type IV–VIII. AHA Type IV–VII showed higher A^wall/T_1_^, SI^wall/T_1_^, and AS^T_1_^ than normal controls (Table 3).

**Fig. 4.**
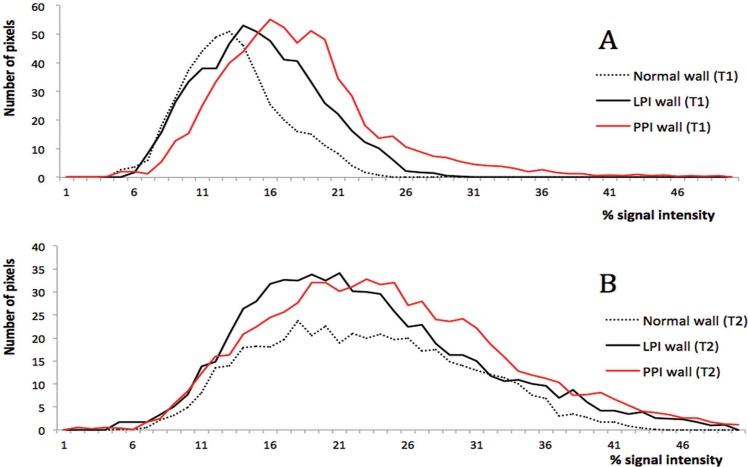
T_1_ (**A**) and T_2_ (**B**) voxel-based histograms (VBHs) of wall of patients with paramedian (red) and lacunar (solid) pontine infarction. On T_1_ VBH for vessel wall (A), the mean was shifted to right side compared with normal control (dot).

**Fig. 5.**
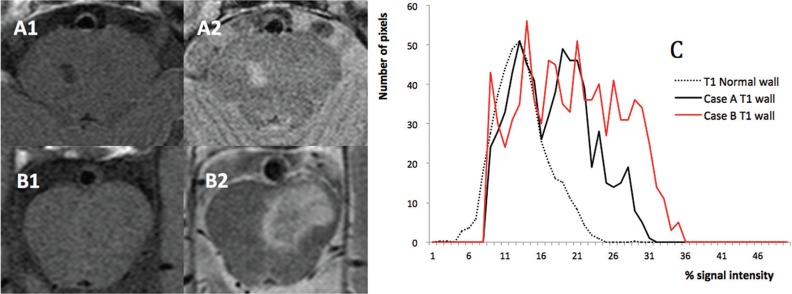
Comparison of T_1_ and T_2_-weighted vessel wall magnetic resonance imaging between a 59-year-old man with lacunar pontine infarction (**A**) and a 68-year-old woman with paramedian pontine infarction (**B**). T_1_ voxel-based histograms (**C**) of paramedian pontine infarction shows increased area (21.12 mm^3^ vs.16.23 mm^3^), higher signal intensity (1.29 vs. 1.00), and right shift of skewness (0.10 vs. 0.23), compared with that of lacunar pontine infarction.

Six AHA Type I & II, 7 AHA Type III, and 3 AHA Type IV–VIII were classified as LPI and 2 AHA Type I & II, 11 AHA Type III, and 13 AHA Type IV–VIII were classified as PPI. Sixteen patients (61.5%) of PPI and four (25.0%) of LPI showed posterior location of plaque. There was no significant difference for TOF MRA between PPI and LPI (Table 4). In comparison of the parameters of VBH between LPI and PPI, A^wall/T_1_^, SI^wall/T_1_^, SK^wall/T_1_^, and A^lumen/T_2_^ showed statistical differences (*P* < 0.05, Kruskal-Wallis test). PPI showed statistical differences in A^wall/T_1_^, SI^wall/T_1_^, SK^wall/T_1_^, and A^wall/T_2_^ than those of normal control after post hoc test, whereas LPI in A^wall/T_1_^ and A^wall/T_2_^. In comparison between PPI and LPI, A^wall/T_1_^, SI^wall/T_1_^, SI^wall/T_1_^, and A^lumen/T_2_^ showed statistical differences (*P* < 0.05, Dunnett T3 procedure) (Table 5; Figs. 4, 5).

## Discussion

These results suggested that VBH of HRMR has the possibility to overcome the shortcomings of preexisting luminographic modalities, by taking into account the arterial wall and lumen at the same time.^19,20^ MR luminographies such as TOF MRA or contrast-enhanced MRA (CE MRA) are clinically useful and easily interpreted for the presence of luminal narrowing.^3,21^ In similarity to conventional angiography, the measurement of stenosis rate can be determined by the gold standard criteria such as European Carotid Stenosis Trial, North American Symptomatic Carotid Endarterectomy Trial. Apart from clinical usefulness, MRA has several pitfalls. First, the accuracy of the measurement of stenosis rate is doubtful since flow-related artifact may result in an apparent reduction in a vessel’s caliber or complete loss of visualization.^21^ Second, it shows minimal diameter reduction or even a normal diameter when atherosclerotic plaque develops mainly toward the outer side. Third, projection image does not visualize the arterial stenosis correctly and stenotic lesions can be hidden depending on image acquisition plane.^22,23^ In addition, the measurement results were dependent on the variability between readers due to different experience or methodology.

In this study, wall area measurement using VBH analysis was well-correlated with the MRI-modified AHA grading, showing good agreements with pathologic progression. From the original AHA classification based on histopathological findings, AHA classification was modified for MRI finding.^18,24,25^ According to AHA classification for MRI, Type I & II are indistinguishable from the near-normal carotid wall. Type III is characterized by a slight diffuse or eccentric thickening of the wall containing pools of extracellular lipid. Therefore, AHA Type III appeared diffuse thickening of wall with slightly high signals on T_1_WI. AHA Type IV–VIII contains various chemical components including lipid or necrotic core, which by MR may have high or iso-signal intensity on T_1_WI, and varied SI on T_2_WI. As advantage over other modalities in correlation with AHA grading, VBH is useful to estimate early atherosclerotic change. In comparison with the normal control group, early stage of atherosclerosis (AHA Type I & II) had increased area of wall and lumen. Even if there were no definite visible plaque or stenosis, early changes of atherosclerosis of the vessels had impact on wall and lumen. These findings also suggest that there is an ongoing adaptation mechanism of atherosclerotic change, namely “positive remodeling.” In comparison with normal control group, luminal areas of intermediate or advanced atherosclerosis (AHA Type III or IV–VII) were not different, which could suggest the lumen was narrowing due to atheroma protruding into the lumen with progression of atherosclerosis. Ma et al.^26^ reported that positive remodeling was more commonly seen in advanced basilar atherosclerosis and positive remodeling lesion had greater luminal area and wall itself with significantly larger plaque than non-positive remodeling lesion.

In addition to wall area measurement, signal intensity measurement could be a promising method to analyze the histology of atherosclerotic plaque. The main components of the atherothrombotic plaques are connective tissue, cholesterol compound, variable cells including monocyte-derived macrophages, T-lymphocytes, and smooth muscle cells. And thrombotic materials with platelets and fibrins are added in advanced or ruptured plaques. Varying proportions of these components are present in different plaques, thus giving rise to heterogeneity of the lesions. Vulnerable plaques, which are prone to rupture, contain a high frequency of inflammatory cells and may cause clinically manifest problems. Each voxel of MRI of atherosclerotic vessels represents various composition of atherosclerotic plaque. We hypothesized the pattern of VBH represent the status of atherosclerotic status quantitatively. As VBH based on pixel intensity of interest region have used to estimate the different component of histology, the skewness and kurtosis of VBH could represent different biochemical component, even though the volume of vessel wall similarly increased. Increased kurtosis (K^wall/T^_1_) was noted in AHA Type I & II, which is believed to reflect increased numbers of pixel from homogeneous wall component. Whereas decreased skewness (SK^wall/T^_1_) was noted in AHA Type IV–VIII, which is probably due to increased number of pixel from abnormal tissues. This study also demonstrated that signal intensities on T_1_WI of PPI subgroup, in addition to wall areas on T_1_- and T_2_WI, are larger than those of the LPI subgroup. A positive arterial adaptation to plaque development, known as “Glagov phenomenon,” explains this high incidence of basilar plaque both in PPI and LPI.^15^ High frequency of PPI occurrence could be related with increased probability atheroma covering or significant narrowing of orifice of basilar artery and embolization of atherosclerosis materials. PPI were more frequent in case with atheroma located in posterior side of basilar artery. Consequently, our methodology showed categorical qualitative progression of intracranial atherosclerosis as well as stenosis degree, measured by numeric data, which could be useful as a screening tool to evaluate intracranial atherosclerotic disease. Although visual assessment of MRI for atherosclerotic vessel has been developed especially for internal carotid artery since Cai et al.,^18^ it could be depended on individual interpreter. Whereas VBH could be reproducible, independent from examiner, and able to detect minimal signal changes of the atherosclerotic vessel.

This study had some limitations that should be considered. First, VBH was obtained from vascular image using a 1.5T MRI, which has limited spatial resolution. The limitation could raise the possibility of a flow-related artifact, which mimics the wall thickening. These artifacts could make considerable discrepancies of area of wall and lumen between T_1_WI and T_2_WI in this study. To make the imaging of vessel wall adjacent to the intravascular space clearer, MR technique for the suppression of signal from blood flow, so-called black-blood imaging, can be applied by alternating echo and repetition time for T_1_WI, and T_2_WI even at 1.5T.^27^ In addition, lumen–intima or adventitia–CSF interface intensities cause considerable variations in measuring area, requiring MRI scale standardization.^28^ Especially, as T_2_WI depicts the boundaries with less clarity, it could not be inappropriate to measure the area of wall. A 3T or higher magnetic field MR machine with optimal flow suppression technique could identify better basilar artery plaques.^14,17^ Second, a relatively small number of patients and normal controls were enrolled in our study, because the incidence of isolated PI was not so high. Therefore, statistical power of this study could be limited. Small number of patients made it impossible to analyze using parametric statistical analysis. We used Kruskal-Wallis test or Mann Whitney U test instead of ANOVA or t-test.

The current study showed that the wall area of basilar artery in patients with isolated PI measured on both T_1_- and T_2_WI increased along with the advanced AHA grading. The mean signal intensity also increased along with AHA grading. However, no differences were found among AHA subgroups, except the stenosis rate, when evaluated from visual assessment of HRMR or TOF MRA. Therefore, the wall area measurement may be the most powerful tools in providing information comparable to histopathological progression of atherosclerotic plaque. Based on these findings, VBH analysis of HRMR could be useful tool to evaluate the progression and the histologic status of atherosclerotic plaque. In conclusion, VBH was useful in the evaluation of atherosclerotic change of basilar arteries in patients with PI. Although it is required to validate the presented VBH for atherosclerotic vessel with optimal MR sequence and post imaging processing program, for use in clinics, the results should serve as the basis for further investigation.

## Figures and Tables

**Table 1.** Demographic data of patients according to the classification of pontine infarction

	Lacunar PI (n = 16)	Paramedian PI (n = 26)	*P* value
Age	57.06 ± 12.03	67.66 ± 11.38	0.004
Sex (Male)	12 (75.0%)	13 (50.0%)	0.195
Hypertension	10 (62.5%)	19 (73.1%)	0.51
Diabetes mellitus	6 (37.5%)	12 (46.2%)	0.750
Cigarette smoking	9 (56.2%)	9 (34.6%)	0.210
Hypercholesterolemia	3 (18.8%)	7 (26.9%)	0.715
GCS	15.00 ± 0.00	14.69 ± 1.05	0.164
Initial NIHSs	2.25 ± 2.21	5.15 ± 4.31	0.025
Duration of admission	11.69 ± 9.81	19.34 ± 14.27	0.262
mRS	0.938 ± 1.24	1.31 ± 1.44	0.393

Brain MRI			
Basal ganglia lacune	6 (37.5%)	12 (46.2%)	0.750
Posterior fossa lacune	3 (18.8%)	4 (15.4%)	1.000

GCS, Glasgow Coma Scale; mRS, modified Rankin Scale; MRI, magnetic resonance imaging; NIHS, National Institutes of Health Stroke Scale; PI, pontine infarction.

**Table 2.** Comparison of visual assessment of vascular MRI, TOF MRA, and transcranial Doppler of basilar artery, according to the MRI-modified AHA atherosclerotic plaque schema

	AHA I & II	AHA III	AHA IV–VIII (C)	*P* value	Post hoc <0.05

	(A, n = 8)	(B, n = 18)	(C, n = 16)		
Visual assessment					
Shortest luminal diameter	2.93 ± 0.11	3.10 ± 0.25	2.79 ± 0.29	0.239	
Longest luminal diameter	3.08 ± 0.11	3.46 ± 0.16	3.44 ± 0.18	0.212	
Outer diameter	4.62 ± 0.15	4.91 ± 0.20	5.45 ± 0.17	0.031	A&C

TOF MRA					
Stenosis (−)	7 (87.5%)	14 (77.8%)	8 (50.0%)	0.284	
Stenosis (+)	1 (12.5%)	4 (22.2%)	6 (50.0%)		

AHA, American Heart Association; TOF MRA, time-of-flight magnetic resonance angiography.

**Table 3.** Comparison of parameters of voxel-based histographic analysis, according to the MRI-modified AHA atherosclerotic plaque schema

	Normal Control	AHA I & II	AHA III	AHA IV–VIII	*P* value	Post hoc <0.05**
	(N, n = 10)	(A, n = 8)	(B, n = 18)	(C, n = 16)		
T_1_WI wall						
Area, mean (mm^3^)	9.38 ± 1.35	11.68 ± 2.36	14.54 ± 2.56	16.96 ± 7.23	0.001	NB, NC
Signal intensity, mean	0.55 ± 0.04	0.65 ± 0.11	0.68 ± 0.07	0.82 ± 0.12	<0.001	NB, NC, AC
Signal intensity, SD	0.14 ± 0.04	0.17 ± 0.11	0.19 ± 0.08	0.20 ± 0.06	0.025	
Skewness	0.64 ± 0.26	0.56 ± 0.53	0.48 ± 0.43	0.14 ± 0.20	0.001	NC, AC
Kurtosis	−0.41 ± 0.34	−0.39 ± 1.67	−0.21 ± 0.99	−0.78 ± 0.69	0.009	BC
Lumen, mean area (mm^3^)	5.45 ± 1.42	8.34 ± 3.45	8.50 ± 4.11	5.90 ± 3.00	0.056	
Areal stenosis*	0.63 ± 0.04	0.59 ± 0.06	0.64 ± 0.11	0.74 ± 0.10	0.002	NC, AC

T_2_WI wall						
Area mean (mm^3^)	11.36 ± 2.66	13.48 ± 2.88	15.18 ± 3.66	19.29 ± 7.54	0.004	NB, NC
SI mean	1.07 ± 0.19	1.07 ± 0.16	1.15 ± 0.19	1.31 ± 0.29	0.038	
SI SD	0.30 ± 0.06	0.31 ± 0.13	0.29 ± 0.08	0.30 ± 0.09	0.864	
Skewness	0.16 ± 0.36	−1.41 ± 0.58	0.15 ± 0.54	0.11 ± 0.35	0.546	
Kurtosis	−0.59 ± 0.46	−0.31 ± 0.47	−0.11 ± 1.46	−0.62 ± 0.40	0.403	
Lumen, mean area (mm^3^)	6.61 ± 2.03	8.87 ± 2.51	10.75 ± 4.48	7.00 ± 7.32	0.029	NB
Areal stenosis*	0.63 ± 0.07	0.60 ± 0.07	0.60 ± 0.09	0.73 ± 0.09	0.002	AC,BC

AHA, American Heart Association; SD, standard deviation; SI, signal intensity; WI, weighted imaging;

* , A^wall^/(A^wall^ + A^lumen^);

** , NB, NC, AC, and BC, statistically significant between N and B, between N and C, between A and C, and between B and C, respectively.

**Table 4.** Comparison of the visual assessment of vascular MRI, TOF MRA, and transcranial Doppler of the basilar artery, according to the classification of pontine infarction

	Lacunar PI	Paramedian PI	*P* value

	(n = 16)	(n = 26)	
Visual assessment			
AHA classification			
I & II	6 (37.5%)	2 (7.7%)	0.009
III	7 (43.8%)	11 (42.3%)	
IV–VIII	3 (18.8%)	13 (50.0%)	
Posterior location of plaque	4 (25.0%)	16 (61.5%)	0.029
Shortest luminal diameter	3.22 ± 0.79	2.80 ± 1.09	0.400
Longest luminal diameter	3.44 ± 0.71	3.35 ± 0.61	0.897
Outer diameter	5.33 ± 0.84	5.69 ± 1.10	0.331
TOF MRA			
Stenosis (−)	13 (81.2%)	18 (69.2%)	0.485
Stenosis (+)	3 (18.8%)	8 (30.8%)	

AHA, American Heart Association; PI, pontine infarction; TOF MRA, time of flight magnetic resonance angiography.

**Table 5.** Comparison of parameters of voxel-based histogram analysis, according to the classification of pontine infarction

	Normal control	LPI	PPI	*P* value	Post hoc <0.05**
	(N, n = 10)	(A, n = 16)	(B, n = 26)		
T_1_WI wall					
Area, mean (mm^3^)	9.38 ± 1.35	13.38 ± 3.26	15.86 ± 5.96	<0.001	NA, NB, AB
Signal intensity, mean	0.55 ± 0.39	0.66 ± 0.14	0.77 ± 0.11	<0.001	NB, AB
Signal intensity, SD	0.15 ± 0.04	0.18 ± 0.08	0.18 ± 0.07	0.077	
Skewness	0.64 ± 0.26	0.47 ± 0.48	0.30 ± 0.37	0.090	NB
Kurtosis	−0.41 ± 0.34	−0.14 ± 1.24	−0.28 ± 0.99	0.781	
Lumen, mean area (mm^3^)	5.45 ± 1.42	8.21 ± 4.15	7.03 ± 3.26	0.130	
Areal stenosis*	0.63 ± 0.04	0.63 ± 0.10	0.69 ± 0.10	0.126	

T_2_WI wall					
Area mean (mm^3^)	11.36 ± 2.66	16.09 ± 4.99	16.62 ± 6.39	0.013	NA, NB
Signal intensity, mean	1.09 ± 0.17	1.08 ± 0.16	1.24 ± 0.26	0.050	AB
Signal intensity, SD	0.30 ± 0.06	0.28 ± 0.12	0.31 ± 0.08	0.612	
Skewness	0.16 ± 0.36	0.05 ± 0.42	0.01 ± 0.54	0.839	
Kurtosis	−0.59 ± 0.46	−0.57 ± 0.46	−0.21 ± 1.24	0.376	
Lumen, mean area (mm^3^)	6.61 ± 2.03	11.75 ± 6.80	9.77 ± 5.65	0.002	NB, AB
Areal stenosis*	0.63 ± 0.07	0.60 ± 0.09	0.60 ± 0.08	0.963	

LPI, lacunar pontine infarction; PPI, paramedian pontine infarction; SD, standard deviation; WI, weighted imaging;

* , A^wall^/(A^wall^ + A^lumen^);

** , NA, NB, and AB, statistically significant between N and A, between N and B, and between A and B, respectively.
